# Development and Validation of Nutrition Environment Scoring for Chinese Style University/Work-Site Canteens (NESC-CC) and Oil–Salt Visual Analogue Scale (OS-VAS)

**DOI:** 10.3390/ijerph192114169

**Published:** 2022-10-29

**Authors:** Yue Han, Zhihong Fan, Yixue Wu, Dandan Zhang, Xinyi Wen

**Affiliations:** College of Food Science & Nutritional Engineering, China Agricultural University, Beijing 100083, China

**Keywords:** nutrition environment, oil and salt, visual analogue scale, Chinese-style canteens

## Abstract

The assessment of the use of cooking oil/fat and salt in dining food is an unsolved issue in non-quantitative nutrition environment evaluation, and the nutrition environment of Chinese-style dining establishments has not been effectively assessed. This study developed two evaluation tools: the Nutrition Environment Scoring for Chinese Style University/Work-site Canteens (NESC-CC) and the Oil–Salt Visual Analogue Scale (OS-VAS) and applied them in nine university canteens. The NESC-CC, which includes comprehensive items from the healthiness of food availability, cooking methods to other factors that support healthy choices, is featured by adjusting the scoring categories and items to suit Chinese food culture and canteen operation model. The OS-VAS is a novel virtual analog scale (VAS) based on the fuzzy judgement of the diners. It requires the randomly recruited respondents to rate their personal preference for salty taste/greasy food preference, overall saltiness/greasiness of canteen dishes, and personal demands for salt/cooking oil reduction. The oil use score, the salt use score, and the total score are derived from the given formula. The field tests of the NESC-CC in nine university canteens at three time points showed that this tool was able to effectively distinguish the nutrition environment of the Chinese food-style canteens with good reliability and validity. The result of OS-VAS scoring achieved a good resolution of the overall salt and oil/fat use and confirmed our hypothesis on the fuzzy judgement of the diners These tools are suitable for the comprehensive evaluation of Chinese-style canteens and have the potential to be applied to more group-meal-providing establishments.

## 1. Introduction

With the development of economy and society and the improvement of living standards, the proportion of people eating out frequently has gradually increased. The resulting unbalanced nutritional intake and unhealthy dietary pattern increases the risk of overweight, obesity, and the related chronic metabolic diseases [1].

Nutrition environment is a conceptual model first proposed in 2005 [2] with a perspective of the possible impact of food environment on personal food consumption. There is evidence that the nutritional environment plays a key role in dietary patterns, food choices, and energy intake [3,4], and thus influences the risk of obesity and chronic diseases. In recent years, many studies on obesity prevention have also focused attention on environmental factors, contributing to the formulation and publication of relevant health policies [5,6,7].

Among the nutrition environment variations, the consumer food environment in catering services is a key factor to be addressed as more and more young people depend heavily on packaged food, take-out food, and all kinds of ready-to-eat food providers. A number of evaluation tools, such as the Nutrition Environmental Measures Survey (NEMS-R) [8], the Freedman’s comprehensive dining survey [9] and the Full Restaurant Evaluation Supporting a Healthy (FRESH) Dining Environment Audit [10], have been developed to assess the availability of healthy or unhealthy food, the means to encourage access to food and the price policy of food, as well as other factors that may impact consumer’s healthy eating decisions in dining occasions.

However, due to the differences in food culture, cooking practices and dietary patterns, the evaluation tools developed based on Western food culture cannot be simply applied to the nutritional environment survey and evaluation in China. The nutrition environment evaluation of Chinese restaurant and canteens can be a great challenge as Chinese cuisine characterized by its diversified food materials, complicated cooking methods and rich food choices. For example, diners have to make choices among more than 30 kinds of dishes, which are made of over 50 raw materials, in just one of the canteens at a university campus. To our best knowledge, the nutrition environment survey of Chinese dining places has not yet been conducted.

In addition to the difficulty in identifying healthy food choices based on many meat–vegetable mixed dishes, the evaluation of nutritional environment of a canteen or restaurant can be further challenged by the need for judging the amount of cooking oil/fat and salt in the dishes. Compared with packaged food, sodium content in restaurant food is more difficult to be assessed and managed [11]. Excessive salt intake or high sodium intake is the main risk factor for hypertension [12,13,14], overweight and obesity [15], osteoporosis, kidney stones and other diseases [13]. It is closely associated with an increased risk of all-cause mortality [16,17] and excessive intake of fat can affect gut microbial homeostasis closely related to metabolism and excess energy accompanied by the abnormal metabolism of sugar, fat and water and salt can cause overweight and obesity, diabetes and cardiovascular disease [18,19,20,21]. Some studies indicated that high-fat and high-salt diets might cause cognitive impairment [22,23].

The World Health Organization has included the reduction of salt/sodium intake in the 2013–2020 global action plan for the prevention and control of non-communicable diseases [24] and on this basis has developed the SHAKE plan (Surveillance, Harness industry, Adopt standards for labeling and marketing, Knowledge, Environment) to promote the implementation of salt reduction strategies [25]. Considering the possible health consequences, there is an urgent need to develop a practical tool to assess the use of cooking oil/fat and salt use in public food service providers.

Since the university students spend most of their time on campus and heavily rely on the food provided by the canteens and cafeteria there, the assessment of the nutrition environment of university canteens is of great significance to improve the nutritional status of the students.

In this study, we developed Nutrition Environment Scoring for Chinese Style University/Work-site Canteens (NESC-CC) and a novel tool for oil and salt added in dining places through the Oil–Salt Visual Analogue Scale (OS-VAS) based on the collective perception of diners. The NESC-CC and OS-VAS were tested and validated in nine canteens of China Agricultural University. This paper describes the development of the above-mentioned two tools and their reliability and validity.

## 2. Methods Overview

The present study consists of two studies. Study 1 is the development and validation of the Nutrition Environment Scoring for Chinese Style University/Work-site Canteens (NESC-CC). Study 2 is the development and validation of the development and application of Oil–Salt Visual Analogue Scale (OS-VAS). The tools were formulated in 2021 and revised three times while tested in twice pilot surveys. The data collection was carried out through April to May 2022 on the campus of China Agricultural University.

The China Agricultural University is a public university in Beijing with about 27,000 undergraduate and graduate students, most of whom live on-campus and whose main dining venue are the nine canteens located inside the campus, which are managed by the university. The canteens provide Chinese-style meals and have daily meal plans. Students choose the staple food and dishes as they like and complete the payment and pick up their meals at the windows. They can either eat in the canteen or as a takeaway. The food sold in the canteen includes staple food, Chinese salad dishes, main dishes, cooked vegetable, fruits and drinks, etc., which are similar to the food that can be obtained at home. In our study, the nine canteens were scored for their nutrition environment and students who dined in the canteens were asked to complete the evaluation of the amount of oil and salt added.

### 2.1. Study 1 Development and Application of Nutrition Environment Scoring for Chinese Style University/Work-Site Canteens (NESC-CC)

#### 2.1.1. The Conception and Structure of the NESC-CC

The goal of the NESC-CC is to build an evaluation tool for the nutrition environment of Chinese canteens. The conception of the tool drew from the conceptual model and evaluation framework of nutrition environment. Factors such as the availability of healthy choices, the modification of food service, the promotion of foods and the information environment that might impact the nutritional environment are included in the NESC-CC scoring items (File S1). In order to evaluate the cooking methods and dining environment of Chinese canteens, we added a part regarding the canteen’s facilities and related services to the framework of the tool.

Regarding the definition and types of healthy food, we applied the relevant content of the Dietary Guidelines for Chinese Residents [26] to the evaluation framework of NESC-CC. In 2005, the Chinese Dietary Balance Index (DBI) [27] proposed the combination of the content of the balanced diet pagoda and the dietary guidelines for Chinese residents and has been applied to the evaluation of dietary quality in many regions of China [28,29,30,31]. DBI evaluates the dietary quality of the population in terms of food type and intake, which is also reflected in the NESC-CC (File S1). In the evaluation module of canteen facilities and related services, we incorporated the requirements of the Guidelines for the Construction of Nutritious and Healthy Canteens [32] into NESC-CC.

#### 2.1.2. Determine Scoring Items

Before forming the final draft, we conducted interviews with canteen leaders to understand the basic situation of each canteen. Subsequently, we revised the scoring content of NESC-CC, adjusting the time and content of the evaluation. In order to make the evaluation more feasible and discriminatory, we added the statistics of the number of food on the basis of the score. After discussion and consensus, we determined the NESC-CC scoring items.

The purpose of developing NESC-CC is to evaluate the nutritional environment of Chinese canteens from the perspective of food and the factors that influence food choice. In the process of determining the scoring items of NESC-CC, we refer to the relevant content of the Dietary Guidelines for Chinese Residents and the Guidelines for the Construction of Nutritious and Healthy Canteens.

The Dietary Guidelines for Chinese Residents is a guideline on food selection and physical activity based on the nutritional needs of the Chinese population and combined with the data from the China Health and Nutrition Survey. It recommends the intake of cereals, potatoes, vegetables and fruits, livestock, poultry, fish, eggs, milk, soybeans and nuts. The determination of the NESC-CC scoring items is also based on the Dietary Guidelines for Chinese Residents, and the healthiness and intake of food are used as an important basis for scoring.

Regarding the intake of cereals and potatoes, the Dietary Guidelines for Chinese Residents recommends that adults between the ages of 18 and 65 consume 200–300 g of cereals per day, including 50–150 g of whole grains and beans and 50–100 g of potatoes. At the same time, daily intake of cereals, potatoes and beans should comprise three types on average and five kinds every week. Therefore, in the NESC-CC scoring section on staple foods, in addition to the type of food, we also include whether the staple food contains potatoes, whole grains and beans and whether its content reaches 20% or 50% to meet the daily needs of the human body as a point of scoring.

The recommended daily intake of vegetables and fruits is at least 300 g and 200–350 g, respectively, and the intake of dark vegetables, such as spinach, broccoli, tomatoes, carrots, and purple cabbage, is also considered. Therefore, we set up scoring items on the availability and quantity of vegetables and fruits.

The Dietary Guidelines for Chinese Residents recommend an average daily intake of 120–200 g of fish, poultry, eggs and lean meat and 300–500 g of fish, 300–350 g eggs and livestock, and 300–500 g poultry per week Considering that Chinese people mainly eat meat at lunch and dinner, we divided daily and weekly intakes into each meal. In addition, soy, nuts and milk can be present in both food and beverage forms in the Chinese diet, so they all appear in different scoring sections.

Different from the Dietary Guidelines for Chinese Residents, the Guidelines for the Construction of Nutritious and Healthy Canteens focus more on the environment. In terms of organization and management, personnel training and assessment, nutrition and health education, catering and cooking and catering services, the Guidelines for the Construction of Nutritious and Healthy Canteens have laid out detailed requirements. With reference to these, we developed scoring items for different aspects to evaluate the nutrition environment in terms of other factors that influence healthy choices.

Finally, we divided NESC-CC into two parts. The first part was to evaluate the healthiness of the ingredients and food content of the canteen according to the recommendations of the Dietary Guidelines for Chinese Residents. The criteria for determining healthy food include whole grains, cooking with less oil and less salt, sugar-free drinks, etc. The second part is to evaluate other factors that support healthy choices, such as services, facilities, etc. (Table 1).

#### 2.1.3. Training for Raters

The raters of the study were the senior undergraduate students and graduate students in the College of Food Science and Nutritional Engineering, China Agricultural University, who have adequate knowledge on food classification, food sensory evaluation, food safety and nutrition. They were trained for 4 h (two 2 h sessions) on the basic concept of nutrition environment, the goal and contents of NSEC-CC, the identification and semi-quantification of each food group in dishes made of multiple food materials and how to address possible confusing occasions in their future scoring. In terms of the scoring content, we explained misleading concepts, such as whole grains, dark vegetables, and common cooking methods. They were required to give a detailed description of the basic situation of the canteens to be evaluated, the method and content of scoring, the time of scoring and the validity of the data. The photo record of each dish in each rating session was sorted according to a coding system. At the same time, we asked the raters to pre-visit and be familiar with the food sales and dining environment of each food outlet in advance before scoring. With the consent of the canteen managers and the premise of ensuring food safety and the order of the canteen, our raters were allowed to enter the food serving area to evaluate the nutrition environment.

#### 2.1.4. Scoring Process

The nutrition environment scoring for canteens was completed from April to May 2022. Each canteen was independently scored twice by two trained raters according to a uniform standard. The scoring process was divided into three parts: (1) review of menu acquired from the canteen manager to examine the availability of diverse healthy foods; (2) on-the-spot investigation to confirm the actual situation of the food in the canteens, including the evaluation and record the food and health-related information provision in each dining place; (3) taking photos of all food and environmental information as sorted records for future reference. The rating points that could not be directly observed were determined by asking the canteen staff.

The complete NESC-CC is detailed in the Supplementary Materials. In the healthiness of ingredients and food content part of the study, we counted the scores and the number of food items for each scoring item and explained the content that is likely to cause scoring differences between raters. In the other factors supporting the healthy choices part, combined with the recommendations of the Dietary Guidelines for Chinese Residents and the documentation requirements of the Guidelines for the Construction of Nutritious and Healthy Canteens, we scored other aspects other than food that might cause differences in nutritional environment scores.

#### 2.1.5. Data Analysis

The intra-class correlation coefficient (ICC) was used to analyze the agreement between raters. The coefficient values of 0.2–0.4, 0.4–0.6, 0.6–0.8 and 0.8–1.0 indicated weak agreement, moderate degree of agreement, strong agreement and very consistent among raters, respectively. The Pearson’s correlation coefficient test was used to analyze the test-retest reliability of the scheme. The coefficient values of 0.2–0.4, 0.4–0.6, 0.6–0.8 and 0.8–1.0 indicated that the two scores were weakly correlated, moderately correlated, strongly correlated and extremely strongly correlated. One-way ANOVA was used to analyze the differences between different time periods in the same canteen and between different canteens in the same time period. *p* < 0.05 was considered significant difference and *p* < 0.01 was considered an extremely significant difference.

### 2.2. Development and Application of Oil–Salt Visual Analogue Scale (OS-VAS)

#### 2.2.1. Scoring Process

The Visual Analogue Scale (VAS) is a straight line with zero and full marks at both ends, the ends of which are defined as the limits of the sensation or response to be measured [33]. VAS is widely used in clinical pain assessment to record patients’ subjective feelings or opinions [34], among which the rating scale with descriptive language showed better accuracy and ease of use [35].

In OS-VAS, we changed the scale and its meaning. The OS-VAS scale ranges from 0 to 100, indicating respondents’ feelings or needs for different rating content. We set scales of 10, 30, 50, 70 and 90 to represent the tipping points of varying degrees of feeling or need. Respondents were finally presented with a scale with descriptive language that was be subjectively rated (See File S2 for the Figures S1–S6).

The OS-VAS scoring tool had been revised twice to pre-test its feasibility and validation power before the survey was carried out during April to May 2022. The minimal number of randomly invited respondents was set to five percent of average daily diners of each canteen based on the interpersonal variance in the pre-test. We asked the respondents to choose their corresponding taste preferences and perception of the saltiness and oil consumption of different canteen dishes in OS-VAS according to their own judgment. The OS-VAS has hints of preference, saltiness, oil consumption and demand for oil reduction and salt reduction represented by different scores. We denoted the scores of personal saltiness preference, oil consumption preference, overall saltiness of canteen dishes, overall oil consumption of canteen dishes, personal salt reduction demand and personal oil reduction demand as S1, O1, S2, O2, S3 and O3, respectively.

#### 2.2.2. Data Analysis

S1 and O1, respectively, indicate the respondents’ acceptance of the amount of salt and cooking oil used in dishes. The higher the score, the more salty or oily dishes the respondents prefer; S2 and O2 represent the respondents’ perceptions of the saltiness and oiliness of the canteen dishes, respectively. The higher the score, the less salt and oil used in the canteen dishes; S3 and O3, respectively, indicate the respondents’ demand for reducing the use of salt and cooking oil in the canteen dishes. The higher the score, the lower the respondents’ demand for reducing salt and oil in the canteen.

The Salt Score (C_S_), Oil Score (C_O_) and Total Score (C) are calculated separately by the following formulas:

C_S_ = (S2 + S3) × S1/(2 × 100) (1)

C_O_ = (O2 + O3) × O1/(2 × 100) (2)

C = [(S2 + S3) × S1 + (O2 + O3) × O1]/(4 × 100)(3)

The higher the C_S_ or C_O_, the more satisfied the consumers are with the use of salt or oil in the canteen. The higher the C, the more satisfied the consumers are with the overall use of oil and salt in the canteen.

The reliability and validity of the scales were tested by reliability statistics and factor analysis and the original data of each canteen was tested for normality by Kolmogorov–Smirnov test. One-way ANOVA, independent samples *t*-test or nonparametric test were used to analyze the differences of scores among canteens and consumers with different tastes. The follow-up analysis method was selected according to the normal test results. If the data were normally distributed and showed homogeneity of variance, the independent sample *t* test was used for statistical analysis of the data; if the data did not meet the normal distribution, the nonparametric test was used for statistical analysis.

All data analysis on NESC-CC and OS-VAS was carried out with SPSS version 22.0.

## 3. Validation of the Tools

### 3.1. Nutrition Environment Score of Canteens

We found that the canteens will replenish or replace food when some food is sold out, which may cause variations in the nutrition environment of the same canteen at different times as well as the ranking of canteens at a given time points. Therefore, we divided the lunch session into three periods and scored the canteens three times according to the healthiness of ingredients and food content, respectively. The results of scoring by time was presented in Table 2.

The results in Table 2 showed that the nutrition environment of different canteens at the same time had significant differences, while the scores of a same canteen at different times differed to varying degrees as well.

With respect to the other factors supporting healthy choices, the scores of the nine canteens were all relatively low, showing no significant differences (Table 3).

The inter-rater and test–retest analysis showed satisfactory consistency, as shown in Table 4. The ICC value is between 0.71–0.99, the test–retest reliability of NESC-CC is high, and the two scoring results show a moderate or more than moderate correlation in nine canteens. The low value of some canteens is due to the fact that the menu of the canteen is updated once a week.

### 3.2. Evaluation of the Oil and Salt Use in Canteens

The average number of diners per meal in each canteen was about 2000, of which 5% of the diners were randomly selected as respondents. A total of 1009 VAS questionnaires were distributed in our study and 981 valid questionnaires were recovered with an effective rate of 97.22%. Totals of 103, 106, 113, 113, 105, 114, 102, 119 and 106 valid questionnaires were collected from the nine canteens, respectively.

The OS-VAS questionnaires showed good reliability and validity. The Cronbach’s α reliability coefficient was 0.778, indicating that OS-VAS has good internal consistency. Based on the combined results of KMO (0.622) and Bartlett’s test (*p* = 6.699 × 10^−35^), the original data of OS-VAS were suitable for factor analysis. The factor loading matrix analysis showed that the loadings of the six items in the corresponding dimensions were all higher than 0.5 (O1, 0.863; S1, 0.813; O2, 0.777; S2, 0.683; O3, 0.814; S3, 0.807), and the scoring items were valid. The analysis results showed that S1 and O1 belong to dimension 1, and the survey content is consumers’ taste; S2, O2, S3 and O3 belong to dimension 2, and the survey content is the consumers’ evaluation of the amount of oil and salt added to the canteen dishes. The factor analysis results were consistent with the dimension division of OS-VAS score items.

The results of the Kolmogorov–Smirnov test showed that the scores of the nine canteens were all in line with the normal distribution, so we used one-way ANOVA to evaluate the differences in the amount of oil and salt used in the canteens. The scores of oil and salt used in the canteens are shown in Table 5. There are significant differences between canteen B and F, C and H and G and I in terms of C. The canteen B and canteen H had the highest Cs, while the canteen G had the lowest. The canteen G had the lowest score of Co, while the canteen H had the highest.

According to the scores of S1 and O1, we divided the respondents of different cafeterias into four categories, salty preference (S1 ≥ 50), non-salty preference (S1 *<* 50), greasy preference (O1 ≥ 50) and non-greasy preference (O1 *<* 50). The scores of the respondents were calculated by different taste preferences. The independent samples t-test was used to compare the scores of respondents with different taste preferences to explore whether there were differences in their evaluation of the oil and salt use in the same canteen. The respondents who preferred a salty taste, which accounted for 67.43% of the total respondents, gave significantly higher marks on the amount of oil and salt used in each canteen than their counterparts who preferred non-salty food did. In terms of preference for different oil levels, the number of respondents who like oily food (51.41%) and those who dislike oily food (48.59%) is basically the same. Respondents who had a preference for greasy food, which accounted for 48.59% of the respondents, also scored higher than the overall rating (Table 6).

## 4. Discussion

The present study presented the first scoring tool, the NESC-CC, as well as a visualized scale scheme to evaluate the nutrition environment in Chinese-style catering service. The preliminary application of the tools showed good reliability and validity and could effectively distinguish the difference in terms of the nutrition environment of university canteens.

The NESC-CC was structured based on the scientific evidence on the relationship of food intake and the risk of non-communicable chronic diseases. The food group associated with the reduced risk of diabetes and cardiovascular diseases, such as whole grains [36] and dark-green leafy vegetables [37], were given more items and scores compared with other food groups. Animal protein and plant protein food are important for maintaining muscles, especially among Asian people, who tend to have less muscle percentage compared to Caucasian people [38]. Milk and soy milk is encouraged, as most people in China have difficulty meeting the dietary reference intake of calcium [39].

It is difficult to assess Chinese food in terms of healthy food choices because of two reasons. First, it is not easy to categorize and quantify each food as multiple base ingredients are included in one dish. Second, the cooking methods of Chinese cuisine are extremely rich and complicated. At the same time, we tried to integrate the evaluation of the cooking method into the score points based on the idea of nutrient density. Cooking methods that utilize less refined oil are encouraged in the scoring system.

In the process of the validation of NESC-CC, we found that there are obvious differences in the nutrition environment of different canteens in the same university, and the nutrition environment scores of the same canteen at different times varied to some extent. In terms of other factors that support healthy choices, the performance of the nine canteens was not excellent, and most of the canteens received less than half of the total score. The low scores could be improved by educating the canteen managers and encouraging them to investigate ways to achieve a better nutrition environment. These results indicated that NESC-CC could identify the differences between the nutrition environment among food provisions with similar styles in a same culture and location.

The scoring system combines the features of both checklist and NEMS-R and integrated the consideration of cooking method. It is has been successfully applied to the context of the Chinese style canteens with a minimum of 4 h training and achieved satisfactory consistency in inter-participant and test-retest analysis. To our knowledge, this is the first report on nutrition environment assessment for Chinese-style catering service.

Compared to some previous reports on university nutrition environment [40,41], we found that Chinese canteens offer a wider variety of food and higher availability of healthy choices. However, some Chinese dishes were criticized for their high content of energy and salt. The popularity of fried food and salty food was reported by a previous quantitative nutrition survey on Chinese university canteens [41]. The National Nutrition and Chronic Disease Report 2020 showed that the average intake of refined cooking oil in China adults is 37.4 g, which is much more than the recommended amount of 25 g [42]. Excess use of cooking oil is not encouraged as it inevitably decreases nutrient density in spite of the fact that the dominant cooking oil in Chinese cuisine are vegetable oils (such as soybean oil, canola oil and peanut oil), which are rich in unsaturated fatty acid. The salt intake is found to rank first among dietary factors associated with cardiometabolic mortality in Chinese adults [43]. Based on these facts, we took oil and salt assessment as priority issues and developed an innovative VAS scheme to make a non-quantitative and low-cost evaluation possible.

The results of OS-VAS analysis showed that there were significant differences in the amount of oil and salt added between the canteens, which was reflected in the scores of C_O_, C_S_ and C values, confirming the effectiveness and sensitivity of the OS-VAS. When we grouped the respondents according to taste preference, we found that there were significant differences in the respondents’ scores on the amount of oil and salt used in the same canteen (*p* < 0.05), which was consistent with our research hypothesis that that consumers with different taste preferences would have different perceptions of the amount of oil and salt used in the same canteen. The innovative research applied a multi-disciplinary approach of visual analog scale and subjective perception of sensory analysis, which provides a new perspective of non-quantitative measurements of cooking oil/fat and salt, which has remained a key issue in nutrition environment evaluation.

This research has its limitations. The validation of the NESC-CC and the OS-VAS was only carried out in nine canteens of one university. We planned to test them in at least 20 canteens of six universities or other group meal providers, but the plan was suspended due to the COVID-19pandemic. The applicability of these two methods of analysis to catering services of different food types and cultures other than Chinese food is yet to be confirmed. Some scoring items and the weight of the score need to be improved according to the actual situation of different geological areas and culture features. The VAS assessment can only make a subjective evaluation of a general picture of oil and salt used in a canteen. More specific and objective evaluation tools needs to be developed and applied in combination with the OS-VAS to better identify the food sources of excess oil and salt.

## 5. Conclusions

In the present study, a NESC-CC tool for a Chinese-style catering food environment and a novel OS-VAS for the evaluation of cooking oil/fat and salt were developed and validated in nine university canteens with satisfying inter-participant and test–retest consistency. The NESC-CC was proven to be effective and practical for the evaluation of healthy food availability, cooking methods and supportive factors conducive to healthy eating, as well as the training and facility aspect. The OS-VAS is the first virtual analog approach for the investigation of cooking oil/fat and salt use. These tool have the potential to be applied to related nutrition environment research issues but still need to be tested and optimized. In future research, we would like to explore the possibility of combining the evaluation of both the objective nutrition environment and the perceived nutrition environment.

## Figures and Tables

**Table 1:** Structure of Nutrition Environment Scoring for Chinese Style University/Work-site Canteens (NESC-CC).

Part	Section	Number of Items	Score Range
1. Healthiness of ingredients and food content	Staple foods	13	0–16
	Vegetables	12	0–16
	Meat, eggs and aquatic products	6	0–7
	Plant-based protein foods	5	0–7
	Fruits	4	0–4
	Dairy and soy beverages	5	0–4
	Other beverages	11	−2–6
2. Other factors that support healthy choices	Service policies	10	0–12
	Information environment	14	−2–12
	Equipment and facilities	10	0–10
	Personnel training and management	11	0–14
Total		101	−4–108

**Table 2:** Score of healthiness of ingredients and food content by time points in nine canteens.

	Canteen A	Canteen B	Canteen C	Canteen D	Canteen E	Canteen F	Canteen G	Canteen H	Canteen I
11:00–11:30	33.00 ± 1.83 ^a^ *	33.50 ± 1.29 ^a^	25.50 ± 3.70 ^c^	32.00 ± 2.94 ^Aab^ **	13.00 ± 2.94 ^e^	29.25 ± 1.26 ^Ab^	18.25 ± 2.06 ^d^	25.00 ± 1.41 ^Ac^	9.50 ± 3.00 ^e^
11:30–12:00	33.00 ± 2.16 ^a^	34.25 ± 0.96 ^a^	24.50 ± 6.14 ^bc^	27.00 ± 2.16 ^ABb^	14.00 ± 0.82 ^d^	27.75 ± 2.75 ^Ab^	21.25 ± 4.72 ^c^	25.00 ± 2.94 ^Abc^	10.25 ± 2.06 ^d^
12:00–12:30	31.75 ± 5.32 ^a^	33.25 ± 0.50 ^a^	21.75 ± 2.63 ^b^	23.00 ± 1.63 ^Bb^	12.25 ± 2.75 ^c^	22.25 ± 1.71 ^Bb^	19.00 ± 7.35 ^b^	17.75 ± 0.96 ^Bb^	10.25 ± 2.06 ^c^

* When the lowercase letters of the means in the same row are different, it is considered that there is a significant difference between the means (*p* < 0.05). ** When the capital letters of the means in the same column are different, it is considered that there is a very significant difference between the means (*p* < 0.01).

**Table 3:** Score of other factors that support healthy choices in nine canteens.

	Canteen A	Canteen B	Canteen C	Canteen D	Canteen E	Canteen F	Canteen G	Canteen H	Canteen I
Service policies	7	7	6	6	6	6	6	6	7
Information environment	3	0	2	4	0	3	3	2	0
Equipment and facilities	9	9	9	9	9	6	5	7	7
Personnel training and management	7	7	7	7	7	6	5	5	5
Total score	26	23	24	26	22	21	19	20	19

**Table 4:** Inter-rater agreement and test–retest reliability of Nutrition Environment Scoring for Chinese Style University/Work-site Canteens (NESC-CC).

	Canteen A	Canteen B	Canteen C	Canteen D	Canteen E	Canteen F	Canteen G	Canteen H	Canteen I
ICC	0.91	0.86	0.91	0.97	0.98	0.99	0.71	0.98	0.94
Pearson’s	0.68	0.59	0.45	0.99	0.65	0.91	0.50	0.89	0.89

**Table 5:** Oil–Salt Visual Analogue Scale (OS-VAS) results of nine canteens.

	Canteen A	Canteen B	Canteen C	Canteen D	Canteen E	Canteen F	Canteen G	Canteen H	Canteen I
C	21.08 ± 9.89 ^abcd^ *	24.08 ± 12.03 ^ab^	19.91 ± 10.71 ^bcd^	22.10 ± 9.52 ^abcd^	21.08 ± 10.38 ^abcd^	19.59 ± 11.81 ^cd^	18.42 ± 11.15 ^d^	24.38 ± 9.88 ^a^	23.75 ± 12.02 ^abc^
C_S_	22.99 ± 12.36 ^ab^	26.86 ± 14.28 ^a^	22.19 ± 12.54 ^ab^	23.81 ± 11.66 ^ab^	23.61 ± 12.51 ^ab^	22.24 ± 13.53 ^ab^	21.23 ± 13.10 ^b^	26.81 ± 11.03 ^a^	26.53 ± 13.51 ^a^
C_O_	19.18 ± 9.90 ^abc^	21.29 ± 11.72 ^ab^	17.62 ± 11.67 ^abc^	20.39 ± 9.13 ^ab^	18.55 ± 10.03 ^abc^	16.94 ± 12.16 ^bc^	15.61 ± 11.08 ^c^	21.94 ± 10.78 ^a^	20.96 ± 11.86 ^ab^

* When the lowercase letters of the means in the same row are different, it is considered that there is a significant difference between the means (*p <* 0.05).

**Table 6:** Difference analysis of Visual Analogue Scale (VAS) scores of consumers by taste preference groups.

Canteens	Tastes	Number of People	VAS	Tastes	Number of People	VAS	*t*	*p*
Canteen A	Preference for more salt	52	27.38 ± 7.928	Preference for less salt	51	14.67 ± 7.25	8.49	<0.05
Canteen B		61	30.33 ± 10.80		45	15.61 ± 7.68	7.80	
Canteen C		71	23.91 ± 9.71		42	13.14 ± 8.81	5.90	
Canteen D		64	27.74 ± 7.16		49	14.74 ± 6.81	9.77	
Canteen E		70	25.54 ± 9.14		35	12.16 ± 6.08	7.83	
Canteen F		75	24.10 ± 11.14		39	10.91 ± 7.46	6.65	
Canteen G		72	21.69 ± 10.67		30	10.57 ± 7.97	5.14	
Canteen H		91	26.58 ± 8.90		28	17.22 ± 9.65	4.77	
Canteen I		74	27.46 ± 11.84		32	15.16 ± 7.10	1.26	
Canteen A	Preference for more grease	26	29.20 ± 8.67	Preference for less grease	77	18.34 ± 8.75	5.48	
Canteen B		41	33.59 ± 10.82		65	18.08 ± 8.35	8.29	
Canteen C		52	26.85 ± 9.70		61	13.99 ± 7.53	7.93	
Canteen D		44	27.78 ± 7.94		69	18.48 ± 8.68	5.74	
Canteen E		46	26.19 ± 9.62		59	17.10 ± 9.18	4.93	
Canteen F		57	25.06 ± 10.90		57	14.11 ± 10.09	5.57	
Canteen G		53	23.29 ± 11.51		49	13.16 ± 7.96	5.13	
Canteen H		79	27.98 ± 8.66		40	17.27 ± 8.20	6.48	
Canteen I		60	28.85 ± 11.91		46	17.10 ± 8.44	5.68	

## Data Availability

The data presented in this study are available on request from the corresponding author.

## Supplementary Materials

The following supporting information can be downloaded at: https://www.mdpi.com/article/10.3390/ijerph192114169/s1, File S1: Nutrition Environment Scoring for Chinese Style University/Work-site Canteens (NESC-CC). File S2: The Oil-Salt Visual Analogue Scale (OS-VAS).
