# Supplementary material for: Development and Validation of Nutrition Environment Scoring for Chinese Style University/Work-Site Canteens (NESC-CC) and Oil–Salt Visual Analogue Scale (OS-VAS)
Source: Int J Environ Res Public Health. 2022 Oct 29;19(21):14169. doi: 10.3390/ijerph192114169 (PMC9658238; doi:10.3390/ijerph192114169)
Supplement: Supplementary file 1 [file ijerph-19-14169-s001.zip › ijerph-1963146-supplementary.pdf]

International Journal of *Environmental Research and Public Health*

MDPI

Supplement File S1 Nutrition Environment Scoring for Chinese Style University/Work-site Canteens (NESC-CC).

Nutrition Environment Scoring for Chinese Style University/Work-site Canteens (NESC-CC)

Date: Scoring period: Canteen: Rater:

Part 1 Healthiness of ingredients and food content

Table S1 Staple foods

Copyright: © 2022 by the authors. Licensee MDPI, Basel, Switzerland. This article is an open access article distributed under the terms and conditions of the Creative Commons Attribution (CC BY) license (https://creativecommons.org/licenses/by/4.0/).

Healthy Choices	Quantity	Score
Total number of staple foods		
Number of staple foods without added oil, salt and sugar	⩾5	1 pt
Number of staple foods containing whole grains, starchy beans and potato/sweet potato (at least 20%)	⩾3<br>⩾6	1 pt<br>2 pts
Rice-based staple foods that contain at least 20% whole grains (including fried rice, rice rolls, etc.)	Yes<br>⩾3	1 pt<br>2 pts
Rice-based staple foods that contain at least 50% whole grains	Yes	1 pt
Porridge with at least 50% whole grains	Yes	1 pt
Pasta such as steamed buns, pancakes, soba noodles, naked oat noodles, and bread that contain at least 20% whole grains	Yes<br>⩾3	1 pt<br>2 pts
Staple foods containing at least 20% starchy legumes	Yes	1 pt
Steamed/boiled whole corn or corn kernels (without added oil, salt, sugar)	Yes	1 pt
Steamed/boiled/roasted sweet potatoes/yams/taros/potatoes (without added oil, salt, sugar)	Yes	1 pt
A "grain basket" that mixes various food categories such as potatoes, whole grains, beans, nuts, etc.	Yes	1 pt
Staple foods containing dairy or soy	Yes	1 pt
Total number of staple food ingredients	⩾5<br>⩾8	1 pt<br>2 pts
Total score		
Range of scores for this section	0–16	

* Soups and drinks with small amounts of grains are not counted.* Fried whole grains or potatoes can not get points.* Foods with more than 5% added sugar, and foods with strong sweetness get no point, but foods with natural sweetness such as sweet corn, sweet potatoes, and water chestnuts can get points.

Table S2 Vegetables

Healthy Choices	Quantity	Score
Total number of dishes		
Total number of dishes with vegetables (at least 50%)	⩾10 or at least 60% of the total number of dishes<br>1 pt	

Number of dishes with at least 80% vegetables	⩾5 1 pt<br>⩾8 2 pts
Number of dishes containing dark green leafy vegetables (at least 50%)	⩾3 1 pt<br>⩾5 2 pts
Number of dishes containing orange/yellow/red vegetables (at least 50%)	⩾2 1 pt
Number of dishes containing fungus, algae vegetables(at least 20%)	⩾2 1 pt
Number of dishes containing tender legume vegetables such as sweet pea, edamame, vegetable broad beans, etc.(at least 20%)	Yes 1 pt
Self-selected boiled/blanched/steamed vegetables (including Mala Tang(spicy hot pot), etc.)	Yes 1 pt<br>⩾3 2 pts
Vegetables mixed with staple food (vegetables added to steamed rice, bibimbap, fried rice) (more than 25% of total weight)	Yes 1 pt
Vegetable salads of Western or Chinese style	⩾3 1 pt
Vegetables claimed to to have reduced amounts of oil/salt	Yes 1 pt<br>⩾3 2 pts
Number of vegetable ingredients (at least 20% of the raw materials)<br>(including beans, algae, fungus and other vegetables)	⩾5 1pt<br>⩾10 2pts
Total score	
Range of scores for this section	0–16

* Soups with a small amount of vegetables can not get points. * Starchy vegetables such as potatoes, sweet potatoes and purple potatoes do not score, but lotus root, water chestnuts and yam can get points. * Fried vegetables can not get points.

Table S3 Meat, eggs and aquatic products

Healthy Choices	Quantity	Score
Number of dishes containing fish/meat/egg (including animal blood and offal, content at least 25%)	⩾5	1 pt
Dishes containing non-fried fish and other aquatic animal products (content at least 50%）	Yes<br>⩾3	1 pt<br>2 pts
Steamed, boiled or roasted chicken/duck/other poultry food (pure meat content 40-100 g)	Yes	1 pt
Red meat including steamed, stewed or roasted beef/mutton/pork (pure meat content 40-100 g)	Yes	1 pt
Egg-containing dishes (at least 25 g eggs)	Yes	1 pt
Dishes containing eggs that are not fried(steamed eggs, boiled eggs or salads containing at least 25 g eggs)	Yes	1 pt
Total score		
Range of scores for this section	0–7	

* Soups that contain only a small amount of meat or eggs do not score. * Fried aquatic products do not score.

Table S4 Plant-based protein foods

Healthy Choices	Quantity	Score
Number of dishes containing plant protein (at least 20%)		

Number of dishes containing non-fried soy products (tofu, dried tofu, yuba, etc.) (at least 20%)	⩾2 1 pt
Number of dishes with non-fried soy protein (at least 50%)	⩾2 1 pt<br>⩾5 2 pts
Number of dishes containing other non-fried plant protein (at least 20%)(including soybean sprouts, chickpeas, kidney beans, peanuts, natto, tempeh etc.)	⩾2 1 pt<br>⩾5 2 pts
Foods containing unfried/unsweetened nuts/oilseeds, including dishes or staple foods (at least 20%)	⩾3 1 pt<br>⩾5 2 pts
Total score	
Range of scores for this section	0–7

* Plant meat substitutes such as textured vegetable protein (TVP) are included in plant protein. * Salads and Chinese salads with plant protein are also included. * Foods containing fried/sweetened nuts or oilseeds do not score.

Table S5 Fruits

Healthy Choices	Quantity	Score
Number of fruit foods		
Fresh fruit available (including cut fruit)	Yes<br>⩾3	1 pt<br>2 pts
Dried fruit available (5–25 g per serving)	Yes	1 pt
A separate window or counter to sell small portions of fresh/dried fruit	Yes	1 pt
Total score		
Range of scores for this section	0–4	

* Salad fruits included. * Drinks containing fruit are not included. * Desserts containing fruit/dried fruit do not score.

Table S6 Dairy and soy beverages

Healthy Choices	Quantity	Score
Number of dairy and soy beverages		
Plain soy milk or soy nut milk	Yes	1 pt
Plain milk (skimmed, low-fat or full-fat, protein content ⩾ 2.9%)	Yes	1 pt
Yogurt (skimmed, low-fat or full-fat, protein content ⩾ 2.9%)	Yes	1 pt
Homemade sugar-free whole grain soy milk(Mixed pulp of soybean, nut, oilseed and multigrain etc.)	Yes	1 pt
Total score		
Range of scores for this section	0–4	

* Beverages in the canteen are not included, and only beverages served in the dining area. * Sweetened homemade beverages with less than 50% milk/soymilk/nut content and added sugar do not score.

Table S7 Other beverages

Healthy Choices	Quantity	Score
Number of other beverages		
Free direct drinking water or boiled water	Yes	2 pts
Free plain soup with grains and beans (rice soup, millet soup, corn soup, mung bean soup, etc.)	Yes	1 pt

Free unsweetened lemonade, barley tea, buckwheat tea, etc.	Yes	1 pt
Free vegetable soup of negelactable content of fat and salt	Yes	1 pt
Unsweetened flower and fruit tea/original tea/black coffee available (chargeable)	Yes	1 pt
100% fruit juice	No point	
Coffee with milk and sugar	No point	
Diet coke/soda with sweeteners	No point	
Sugar-sweetened beverages including fruit drinks and sweetened flavored milk tea drinks	Yes	−1 pt
Alcoholic beverages	Yes	−1 pt
Total score		
Range of scores for this section	−2–6	

* Beverages from the canteen are not included, only those served in the dining area. * Grain soups are limited to very thin types, with more dry matter being graded as porridge. * Soups that are distinctly salty or sweet do not score.

Total for Part 1:		
Range for Part 1:		−2–60

Part 2 Other factors that support healthy choices

Table S8 Service policies

	Health-Related Services	Score	
Combos	Combos with different portion sizes/calories	Yes	1 pt
	Portion size reduction of staple foods on request	Yes	1 pt
	Extra supply of vegetables on request	Yes	1 pt
Dishes	Half portion choices without extra charge	Yes	2 pt
Staple foods	Portion size reduction on request	Yes	1 pt
Steamed buns, cakes, rice noodles, pancakes, etc.	Small portion available/portion size can be reduced	Yes<br>⩾3	1 pt<br>2pts
Porridge	Optional no added sugar/salt	Yes	1 pt
Soy milk/nut milk	Optional no added sugar	Yes	1 pt
Salad/Chinese salad/noodles/set meal etc.	Low-fat or low-salt dressings available	Yes	1 pt
	Amount of salad dressing/sauce adjustable on request	Yes	1 pt
Total score			
Range of scores for this section		0–12	

Table S9 Information environment

	Information that May Affect Choices	Score	
Dishes	A special window/promotion for fat-reduced dishes	Yes	1 pt
	A special window/promotion for salt-reduced dishes	Yes	1 pt
Staple foods	Promotions for small servings of staple foods	Yes	1 pt
	Promotions for staple foods containing whole grains	Yes	1 pt
Combos	Promotions for nutritious combos	Yes	1 pt
	Calorie reduction combos/fat reduction and muscle gain combos	Yes	1 pt
	Example of a combo made of assorted healthy food	Yes	1 pt
Food nutrition information	Calorie/nutrient information available over the sales window/canteen area	Yes	1 pt

	Calorie/nutrient information available on webpages/mini-programs	Yes	1 pt
Health advice	Advice on healthy eating/nutrition balance available	Yes	1 pt
	Advice on food waste reduction available	Yes	1 pt
Healthy eating advocacy	Publicity of Dietary Guidelines or correct nutritional knowledge	Yes	1 pt
Wrong information	Incorrect health/diet information	Yes	−1 pt
	Unscientific food advertisements/promotions	Yes	−1 pt
Total score			
Range of scores for this section		−2–12	

Table S10 Equipments and facilities

Type of Facilities	Equipments	Score	
Cooking facilities	There are enough steamers to make steamed dishes in addition to steaming rice	Yes	1 pt
	There are electric ovens for making roasted dishes without added fat	Yes	1 pt
	Microwave oven available for heating food	Yes	1 pt
	There are other cooking appliances that facilitate cooking with less oil	Yes	1 pt
Quantitative tools	There are utensils to help with healthy eating, such as healthy plates, rationing spoons, etc.	Yes	1 pt
	Weighing scale available for easy understanding of food portions	Yes	1 pt
	There are health testing equipment, such as weight scale, body fat scale, blood pressure monitor, body mass index calculator, etc.	Yes	1 pt
Cleaning utensils	There are facilities for diners to wash their hands and provide sanitizers, etc.	Yes	1 pt
Garbage classification	There are kitchen waste classification measures and careful implementation	Yes	1 pt
Temperature control facilities	There is air conditioning to ensure a comfortable dining temperature	Yes	1 pt
Total score			
Range of scores for this section		0 to 10	

Table S11 Personnel training and management

Items	Details	Score	
Nutrition manager equipped	At least 1 full-time registered dietitian available	Yes	3 pts
	Equipped with at least 1 trained full-time registered dietitian	Yes	2 pts
	At least 1 part-time registered dietitian	Yes	1 pt
	At least 1 trained non-registered part-time nutrition instructor	Yes	1 pt
Nutrition manager training	Regular training program by nutrition related professionals	Yes	1 pt
Management system	Meal committee consists of diners (staff, teachers and students)	Yes	1 pt
	Evaluation machanism for scoring and grading of nutrition and food safety quality	Yes	1 pt
Communication and interaction	There is a system for regular communication between food providers and diners to improve the quality of food and beverages	Yes	1 pt

	Participatory activities that encourage diners to eat healthy foods	Yes	1 pt
Nutrition service capacity	Having the ability to answering the questions and meet the demends on healthy eating of the diners	Yes	1 pt
Dishes development ability	There are healthy dishes development activities every quarter	Yes	1 pt
Total score			
Range of scores for this section		0–14	

Total for Part 2:		
Range for Part 2:		−2–48

Supplement File S2: The Oil-Salt Visual Analogue Scale (OS-VAS)

Dear diners：

Thank you very much for participating in our survey. This research is a non-commercial public health study, which aimed to explore a new approach to evaluation the use of cooking oil and salt in canteens based on the diner's perception. Please read the explanation given below and mark the scoring scales corresponding to each scoring item according to your real daily experience of dining. Since this is an anonymous survey, your do not need to attach any personal information in the survey.

The OS-VAS rated each of the 6 items (personal saltiness preference, oil consumption preference, overall saltiness of canteen dishes, overall oil consumption of canteen dishes, personal salt reduction demand, and personal oil reduction demand) subjectively, as shown in *Figure 1*. We calibrated each scale and tell the respondents how to mark the scale according to their sensory perception. We measured the distance of the marked location and the "0" origin of each scale as the value of score. The subjective perception of oil and salt use in a canteen is judged by calculating the average score of all respondents.

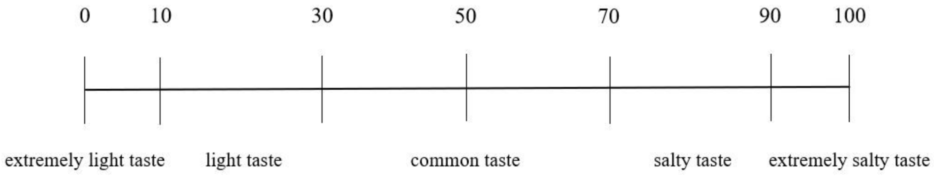

Figure S1. VAS of personal saltiness preference (S1).

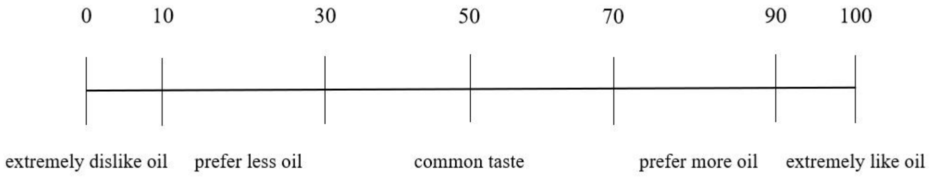

Figure S2. Personal oil consumption preference score, O1.

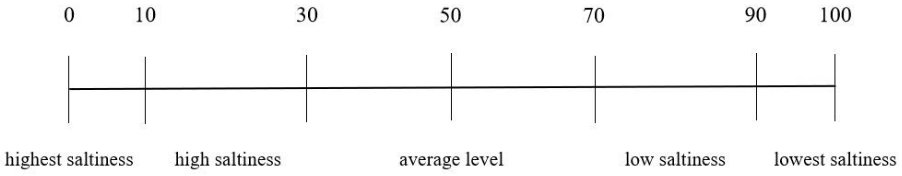

Figure S3. Overall salinity score of canteen dishes, S2.

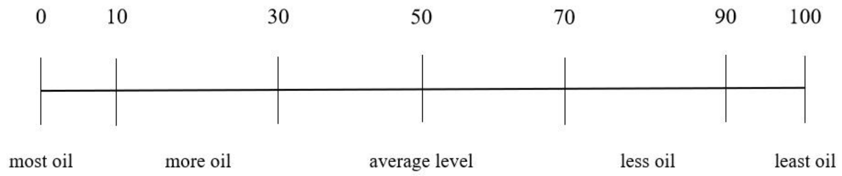

Figure S4. Overall oil consumption score of canteen dishes, O2.

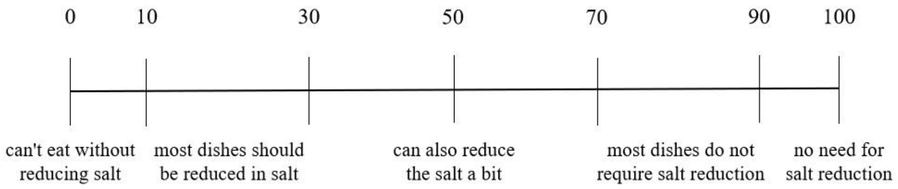

Figure S5. Score of salt reduction demand of canteen dishes, S3.

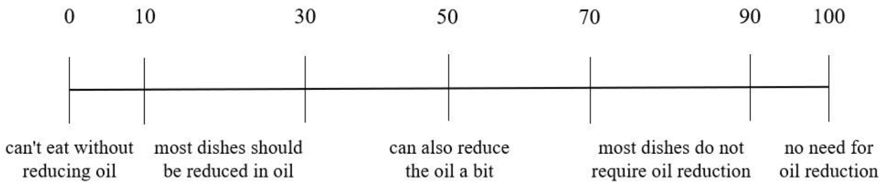

Figure S6. Score of oil reduction demand of canteen dishes, O3.

Thank you for your cooperation, Your scoring will provide very important help to our survey results. If you are interested in the result of the survey, please send your e-mail to hanyuecau@163.com . You are welcome to the food nutrition laboratory in the Food Science Building for more information about our study. Have a nice meal!
